# Landscape and Dynamics of the Transcriptional Regulatory Network During Natural Killer Cell Differentiation

**DOI:** 10.1016/j.gpb.2020.12.003

**Published:** 2020-12-30

**Authors:** Kun Li, Yang Wu, Young Li, Qiaoni Yu, Zhigang Tian, Haiming Wei, Kun Qu

**Affiliations:** 1 Department of Oncology, The First Affiliated Hospital of USTC, Division of Molecular Medicine, Hefei National Laboratory for Physical Sciences at Microscale, School of Basic Medical Sciences, Division of Life Sciences and Medicine, University of Science and Technology of China, Hefei 230021, China; 2 CAS Center for Excellence in Molecular Cell Sciences, The CAS Key Laboratory of Innate Immunity and Chronic Disease, University of Science and Technology of China, Hefei 230027, China; 3 School of Data Science, University of Science and Technology of China, Hefei 230026, China

**Keywords:** NK cell, ATAC-seq, Programmed differentiation, FOSL2, EGR2, Dynamic regulatory network

## Abstract

Natural killer (NK) cells are essential in controlling cancer and infection. However, little is known about the dynamics of the transcriptional regulatory machinery during **NK cell** differentiation. In this study, we applied the assay of transposase accessible chromatin with sequencing (**ATAC-seq**) technique in a home-developed *in vitro* NK cell differentiation system. Analysis of ATAC-seq data illustrated two distinct transcription factor (TF) clusters that dynamically regulate NK cell differentiation. Moreover, two TFs from the second cluster, FOS-like 2 (**FOSL2**) and early growth response 2 (**EGR2**), were identified as novel essential TFs that control NK cell maturation and function. Knocking down either of these two TFs significantly impacted NK cell differentiation. Finally, we constructed a genome-wide transcriptional regulatory network that provides a better understanding of the regulatory dynamics during NK cell differentiation.

## Introduction

Natural killer (NK) cells are innate lymphocytes that survey the environment and protect the host from infected or cancerous cells. As their name implies, NK cells represent an early line of defense during pathogen invasion by directly killing infected cells and secreting inflammatory cytokines [1]. Additionally, NK cell-based immunotherapy has become an emerging force in cancer treatment and will play an essential role in the future treatment [2], [3], [4], [5], [6]. For instance, adoptive NK cell immunotherapy has become increasingly popular due to its capability to induce graft-versus-leukemia effects without causing graft-versus-host disease in patients [7]. Therefore, major efforts are currently underway to fully utilize the anti-tumor properties of NK cells in the clinic. In addition, a variety of methods have been implemented to expand NK cells, establish a microenvironment that favors NK cell activity, and redirect the activity of NK cells onto tumor cells [8]. On the other hand, many researchers use umbilical cord blood (UCB) CD34^+^ cells to produce abundant NK cells [9], [10], [11], [12], [13], [14], [15], [16], [17], [18] for clinical application without feeding cells by adding various cytokines [10]. In our previous work, we have developed a method to obtain sufficient functional NK cells by simply adding a mixture of cytokines and provide a mechanism by which NK cells can be used to treat leukemia [19]. The use of NK cells for immunotherapy relies on the presence of a great number of NK cells with optimal cytotoxic activity [4]; therefore, a comprehensive understanding of the regulatory circuits during NK cell differentiation is particularly important for boosting the efficacy of clinical treatments. However, the mechanisms underlying NK cell differentiation are not well understood. Studies have shown that transcriptional factors (TFs) play an essential role in driving NK cell maturation, and many TFs have been well studied in this process [1]. Additionally, it is known that different TFs play various roles at distinct stages of differentiation [1]. For example, PU.1 is a TF that is known to drive hematopoietic stem cell (HSC) differentiation into the earliest myeloid and lymphoid progenitors [20], whereas T-bet is an essential TF in the control of NK cell maturation and IFN-γ secretion [21]. However, how TFs work in concert to enforce the NK cell phenotype is not clear.

Assay of transposase accessible chromatin with sequencing (ATAC-seq), a newly developed epigenomic profiling technique [22], has been widely used to profile the epigenetic landscapes of cells at specific stages of interest and thereby delineates the underlying regulatory mechanisms of gene expression. For example, previous reports using ATAC-seq identified an exhaustion-specific enhancer that regulates PD-1 expression, thereby elucidating the regulatory mechanism of gene expression in exhausted CD8^+^ T cells [23], [24]. Alternately, ATAC-seq analysis of pure cancer cell populations of human small cell lung cancer (SCLC) identified a novel TF, nuclear factor I B (NFIB), which is necessary and sufficient to drive the metastatic ability of SCLC cells [25]. ATAC-seq has also enabled researchers to track the epigenomic state changes in patient-derived immune cells [26], and to survey how the personal regulomes of the cutaneous T cell lymphoma patients determine their responses towards histone deacetylase inhibitor (HDACi) anti-cancer drugs [27]. More recently, ATAC-seq was applied to better understand the control of NK cells in innate immune memory during infection [28], illustrating the importance of the topic as well as the power of the technique used in this study.

Here, we have developed a systematic method to characterize the chromatin accessibility and regulatory network dynamics during NK cell differentiation from UCB CD34^+^ HSCs based on ATAC-seq. Motif and enrichment analyses from the Hypergeometric Optimization of Motif EnRichment (HOMER) [29] and Genomica [27] show that many TFs play important roles during NK cell differentiation. By integrating gene expression profiles from our previous study [19], two novel TFs, FOS-like 2 (FOSL2) and early growth response 2 (EGR2), were identified to be essential to drive NK cell maturation.

## Results

### Landscape of DNA accessibility during NK cell differentiation

**Figure 1:**
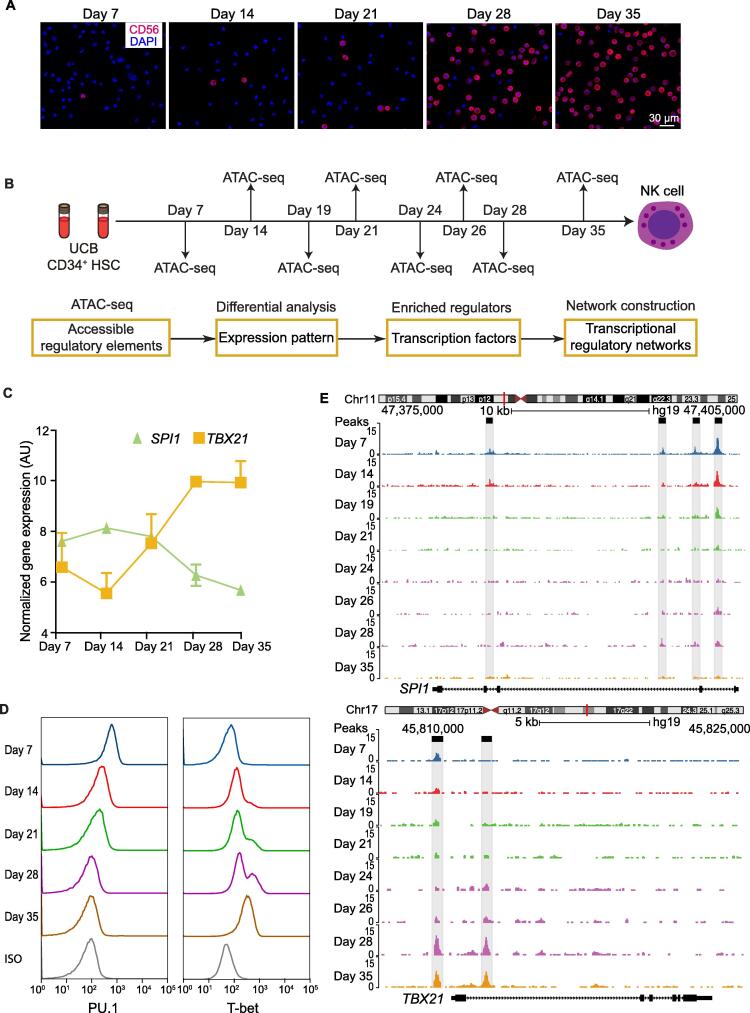
**DNA accessibility during NK cell differentiation** **A.** Confocal microscopy images of membrane CD56 (red) in the cultured cells at day 7, day 14, day 21, day 28, and day 35. Scale bar, 30 µm. Nuclei are stained with DAPI. **B.** Schematic representation of the overall experimental design of this study. Chromosome opening at different time points were assessed using ATAC-seq data. The bioinformatics pipeline for data analysis is shown in the bottom. **C.** The gene expression profiles of *SPI1* and *TBX21* at different stages of NK cell differentiation. **D.** Flow cytometric measurement of PU.1/SPI1 and T-bet/TBX21 expression in cultured cells during a 35-day time course. The X-axis indicates the fluorescence intensity, and the Y-axis indicates the cell number density. **E.** Normalized ATAC-seq profiles of the *SPI1* (top) and *TBX21* (bottom) gene loci at different stages during NK cell differentiation. ATAC-seq signals were obtained from the UCSC Genome Browser. NK, natural killer; UCB, umbilical cord blood; HSC, hematopoietic stem cell; ATAC-seq, assay of transposase accessible chromatin with sequencing; SPI1, Spi-1 proto-oncogene; TBX21, T-box transcription factor 21; AU, arbitrary unit; ISO, isotype control.

To elucidate the regulatory networks during NK cell differentiation, we developed a culture procedure to obtain differentiated NK cells from UCB CD34^+^ cells using a cocktail of cytokines [19]. The differentiation process took 35 days. Interestingly, after culturing of cord blood stem cells for 3 weeks, the proportion of NK cells rapidly increased from 5% on day 21 to approximately 60% on day 28, and peaked at close to 100% on day 35 (Figure 1A, Figure S1A) [19]. To elucidate the dynamic changes of transcriptional regulation during NK cell differentiation, we interrogated the landscape of chromatin accessibility using ATAC-seq at 8 different time points, with 2 replicates each along the process (Figure 1B). Multiple bioinformatics analyses (see Materials and methods) were then performed to obtain the differentially accessed chromatin sites, enriched TFs, and genome-wide regulatory elements. At least 50,000 cells were obtained from each sample, resulting in more than 78 million reads on average with a total of 1260 million reads for all time points (Table S1). From this dataset, 143,570 accessible DNA sites were identified (*P* < 10^−7^, FDR < 10^−7^). Transcription start site (TSS) enrichment score and ATAC-seq signal analysis indicated that the dataset was of high quality with a strong signal-to-background noise ratio and expected fragment length distribution (Figure S1B–E). Moreover, the dynamics of the chromatin accessibility around the known surface marker genes were consistent with changes of their gene expression levels during NK cell differentiation (Figure S1D) [30], [31]. Pearson correlation analysis on the biological replicates at each time point also showed that our ATAC-seq profiles were highly reproducible (Figure S2A and B). Within all the accessible sites, only a very small portion (6.48%) were conserved across all stages (Figure S3A), while the majority of peaks changed over time, suggesting significant chromatin dynamics during NK cell differentiation. Peaks that were detected at all stages were enriched in Gene Ontology (GO) terms such as RNA transcription and other functions to maintain basic physiological activities (Figure S3B). In contrast, more and more immune response-related GO terms were enriched as the time went by, suggesting the activation of critical genes that govern NK cell function.

Several TFs are known to regulate NK cell differentiation, such as PU.1/Spi-1 proto-oncogene (SPI1) and T-bet/T-box transcription factor 21 (TBX21). The former is a NK cell repressor and the latter an activator. Gene expression profiles from our previous study (GEO: GSE87787) [19] indicated down-regulation of the *SPI1* gene and up-regulation of the *TBX21* gene during NK cell differentiation (Figure 1C). The levels of proteins encoded by these two genes were evaluated using flow cytometry, which suggested the same trends (Figure 1D). ATAC-seq analysis revealed four peaks at the intronic enhancers of *SPI1* and two peaks at the promoter and intronic enhancer of *TBX21*, respectively (Figure 1E). Accessible chromatin sites around the *SPI1* gene clearly became unavailable and those around the *TBX21* gene gradually became available during the process, supporting the notion that the epigenetic dynamics of key regulators are consistent with their corresponding gene and protein expression levels. The consistency between the epigenetic and gene expression profiles of TFs known to drive NK cell differentiation, such as GATA binding protein 3 (GATA3) [1] and eomesodermin (EOMES) [1], is shown in Figure S1E, indicating the feasibility of predicting transcriptional regulatory networks from ATAC-seq profiles.

### Epigenomic signatures of different stages during NK cell differentiation

**Figure 2:**
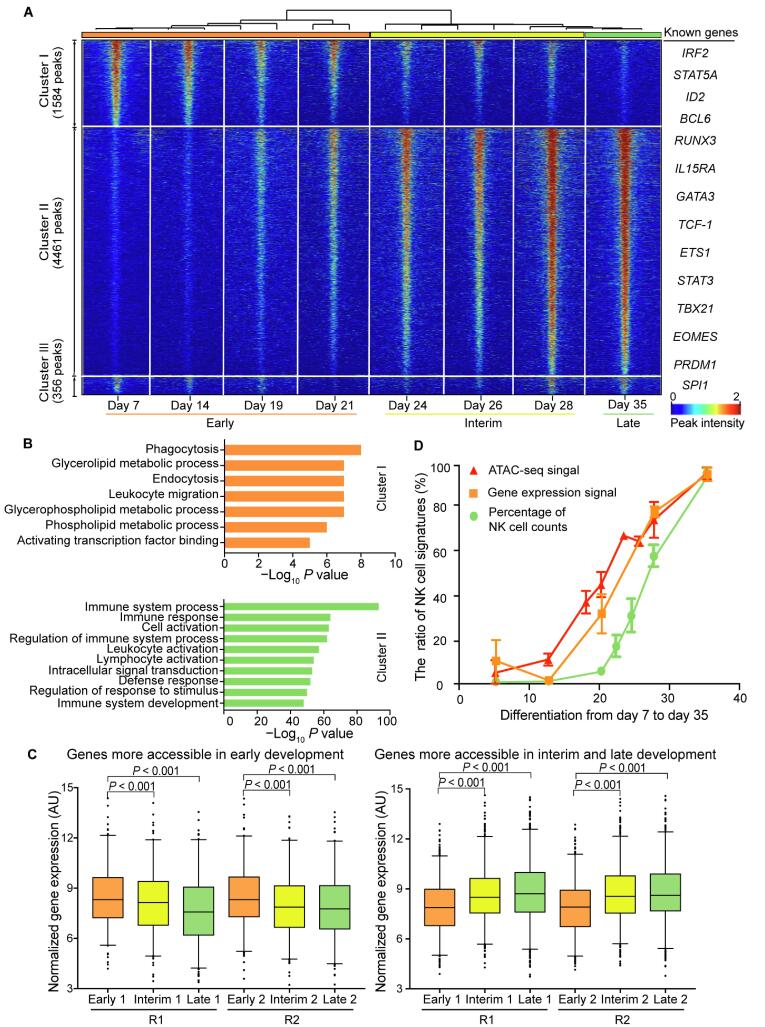
**Differential epigenetic regulation elements during NK cell differentiation** **A.** Heatmap of 6401 differentially accessed regulatory elements during NK cell differentiation. Each column is a sample, and each row is a peak. Samples and peaks are organized by two-dimensional unsupervised hierarchical clustering. Color scale shows the relative ATAC-seq peak intensity centered by each peak summit. Bottom: samples at all time points are categorized into three groups: early stage (days 7–21; orange), interim stage (days 24–28; yellow), and late stage (day 35; green). Samples from the same stage are labeled with the same color. Left: differential peaks are categorized into three clusters. **B.** The top 10 most significant GO terms enriched in cluster I (upper panel) and cluster II (lower panel) peaks. **C.** Box plots showing expression levels of genes in different clusters during NK cell differentiation. Left: genes in cluster I show higher expression in the early stage. Right: genes in cluster II show higher expression in the interim and late stages. R1 and R2 represent biological replicates 1 and 2, respectively**.***P* values are estimated from one-way ANOVA. **D.** The changes in ATAC-seq signal (red), gene expression signal (orange), and percentage of NK cell counts (green) at different time points during NK cell differentiation.

To determine the differences in regulatory DNA activity among different stages during NK cell differentiation, we performed pairwise comparisons of the ATAC-seq signals between the corresponding samples, together with intrinsic analysis [32], a method that highlights the elements with varied accessibility across individuals but not between replicate samples from the same individual (File S1). We discovered 6401 peaks showing differentially accessed DNA sites across the genome, which were categorized into three distinct clusters via unsupervised hierarchical clustering (Figure 2A, Figure S4A and B). Principal component analysis (PCA) of all samples also illustrated three distinct clusters, which is consistent with the time process of NK cell differentiation representing the early, interim, and late stages of the entire process (Figure S4C). GO analysis of these peaks was performed in the Genomic Regions Enrichment of Annotations Tool (GREAT) [33]. Cluster I comprises 1584 elements that are more accessible at the early stage (days 7–21) of differentiation. Peaks in cluster I are significantly enriched in the GO terms of metabolic processes that are necessary for cell viability, proliferation, and differentiation (Figure 2B, upper panel; 10^−8^ < *P* < 10^−4^). Cluster II comprises 4461 elements that are highly accessible at the interim and late stages (days 24–35) of NK cell differentiation. GREAT analysis revealed that peaks in cluster II are strongly enriched (*P* < 10^−50^) in immune-relevant GO terms, such as immune system process, immune response, and immune system development, among others (Figure 2B, lower panel), indicating that the epigenetic states of functional immune cell-specific genes were activated throughout the process. Cluster III consists of only 356 peaks, with no enrichment for GO terms, and were therefore not included for downstream analyses.

We next examined whether the DNA accessibility signatures at different stages are correlated with the expression profiles of corresponding genes. By comparing the ATAC-seq profiles with the genome-wide microarray data during NK cell differentiation, we found that genes that gained chromatin accessibility (cluster II) showed significant increases in expression (Figure 2C, right panel; *P* < 0.001), while genes that lost chromatin accessibility (cluster I) had decreased expression (Figure 2C, left panel; *P* < 0.001), indicating a high correlation between epigenetic and RNA profiles.

Chromatin structures and epigenetic modifications are known to regulate gene expression [34]. However, the chronological order of the dynamics of the chromatin accessibility, gene expression, and cell phenotype has not yet been well studied. Here, we integrated the ATAC-seq signals, microarray profile, and percentage of NK cell counts to examine the temporal dynamics of these three features. Interestingly, we noticed that the accessibility of mature NK cell-specific peaks (cluster II in Figure 2A) started to increase on day 14, the expression of NK cell-specific genes was turned on approximately two days later, while the percentage of NK cell counts started to increase after day 21 (Figure 2D). These results suggest a clear chronological order of the changes in chromatin structure, gene expression, and cell population during NK cell differentiation.

### TF occupancy network during NK cell differentiation

**Figure 3:**
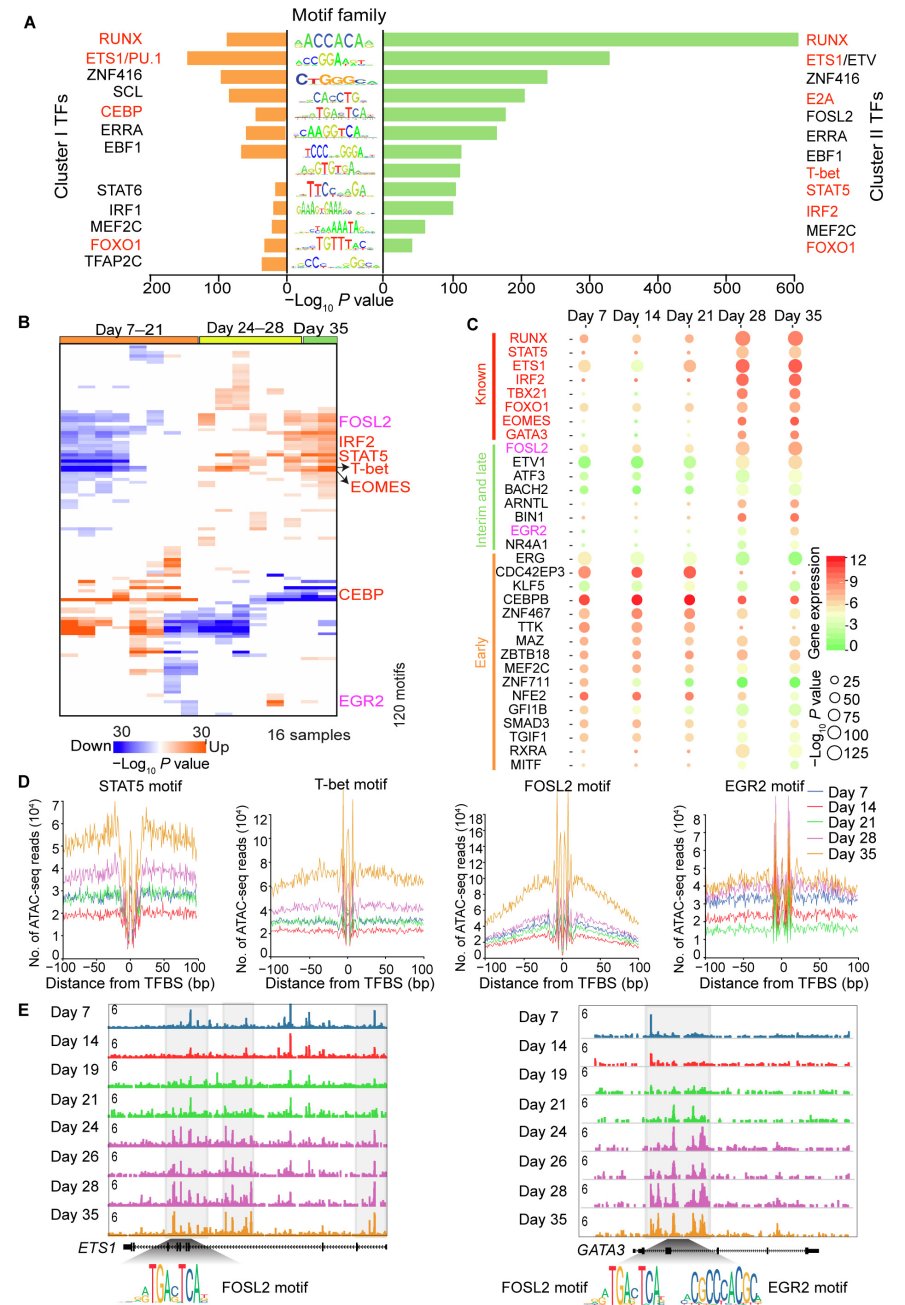
**T**F**s enriched at different stagesof NK cell differentiation** **A.** TF motifs enriched in cluster I (left) and cluster II (right) peaks, with enrichment *P* values estimated from HOMER. TFs known to regulate NK cell differentiation are colored in red. **B.** Enrichment of known TF motifs in differentially accessible elements in all samples. Each row is a TF motif and each column is a sample. Values in the matrix represent the significance levels expressed as –log_10_*P* value of the enrichment estimated from Genomica. Red in the matrix indicates that the motif is enriched in the corresponding sample, whereas blue in the matrix indicates depletion. Red texts on the right indicate known key TFs that regulates NK cell development, while pink texts indicate new TFs whose functions will be experimentally tested later (see Figure 4). **C.** Prediction of TFs that may regulate NK cell differentiation. TFs known to regulate NK cell differentiation are shown at the top, TFs predicted to regulate the early stage of the differentiation process are shown in the bottom, and those predicted to regulate the interim and late stages are shown in the middle. The color of each circle represents the expression level of the gene encoding the corresponding TF, while the size of the circle represents the significance of the motifs estimated by *P* values. **D.** Visualization of the ATAC-seq footprints for STAT5, T-bet, FOSL2, and EGR2 motifs at five indicated time points during NK cell differentiation. ATAC-seq signals across all these motif binding sites in the genome were aligned on the motif and averaged. **E.** Normalized ATAC-seq profiles of the *ETS1* (left) and *GATA3* (right) gene loci at indicated time points during NK cell differentiation. ATAC-seq signals were obtained from the UCSC Genome Browser. The gray blocks indicate genomic loci that are gradually more accessible during NK cell differentiation. The locations of binding motifs for FOSL2 and EGR2 are depicted at the bottom. RUNX, Runt-related transcription factor; ETS1, ETS proto-oncogene 1; ETV, ETS variant transcription factor; ZNF416, zinc finger protein 416; SCL, TAL bHLH transcription factor 1; E2A, E2A immunoglobulin enhancer binding factors E12/E47; CEBP, CCAAT enhancer-binding protein; FOSL2, FOS-like 2; ERRA, estrogen related receptor alpha; EBF1, EBF transcription factor 1; STAT, signal transducer and activator of transcription; IRF, interferon regulatory factor; MEF2C, myocyte enhancer factor 2C; FOXO1, forkhead box O1; TFAP2C, transcription factor AP-2 gamma; EOMES, eomesodermin; EGR2, early growth response 2; GATA3, GATA binding protein 3; ATF3, activating transcription factor 3; BACH2, BTB domain and CNC homolog 2; ARNTL, aryl hydrocarbon receptor nuclear translocator like; BIN1, bridging integrator 1; NR4A1, nuclear receptor subfamily 4 group A member 1; ERG, ETS transcription factor ERG; CDC42EP3, CDC42 effector protein 3; KLF5, Kruppel like factor 5; CEBPB, CCAAT enhancer binding protein beta; ZNF467, zinc finger protein 467; TTK, TTK protein kinase; MAZ, MYC associated zinc finger protein; ZBTB18, zinc finger and BTB domain containing 18; ZNF711, zinc finger protein 711; NFE2, nuclear factor, erythroid 2; GFI1B, growth factor independent 1B transcriptional repressor; SMAD3, SMAD family member 3; TGIF1, TGFB induced factor homeobox 1; RXRA, retinoid X receptor alpha; MITF, melanocyte inducing transcription factor; TFBS, TF binding site.

TFs bind to cognate DNA sequences with patterns termed motifs. Therefore, we could predict TF occupancy on chromatin using DNA accessibility data from ATAC-seq for constructing regulatory networks [26]. To identify potential drivers of NK cell differentiation, we screened TFs that were enriched at accessible elements in clusters I and II using HOMER in the search modes with default (Figure 3A) or setting background (Figure S5A). Our finding suggests that NK cell differentiation and maturation require a variety of TFs. TFs enriched at cluster I peaks are potential regulators at the early stage of NK cell differentiation, while those enriched at cluster II peaks could be critical regulators at the interrim and late stages. We found several TF families that were significantly enriched (*P* < 10^−10^). Many of them are known to be important for NK cell differentiation, such as the Runt-related transcription factor (RUNX) families, ETS proto-oncogene (ETS) families [20], [35], and CCAAT enhancer-binding protein (CEBP) families [36], supporting the reliability of this method to detect critical regulators. For instance, PU.1 is widely expressed and controls multiple stages of bone marrow and lymphocyte differentiation in a variety of hematopoietic-derived lineages [37], [38], [39], [40], [41]. An decrease in the number of NK-cell progenitor (NKP) and immature NK (iNK) cells has been detected in chimeric mice [20], indicating that PU.1 may play an important role during the early stage of NK cell differentiation. Several known TFs, such as RUNX [35], E2A immunoglobulin enhancer binding factors E12/E47 (E2A) [42], T-bet [21], signal transducer and activator of transcription 5 (STAT5) [43], and EOMES [21], are also enriched at the interrim and late stages. The most enriched TFs at cluster II peaks belong to the RUNX family: RUNX1, RUNX, and RUNX2 (Figure S5A), which are key regulators of lymphocyte lineage-specific gene expression [44].

Motif search of differential ATAC-seq peaks could provide information about the transcriptional regulatory network. However, one caveat of this method is the inability to distinguish similar binding motifs of TF family members. Thus, we sought to apply the Genomica’s module map algorithm and “TF footprint” analysis to better predict TF occupancy on accessible sites by integrating the ATAC-seq and gene expression microarray profiles (Figure 3).

In total, 242 vertebrate TF motifs were obtained from the JASPAR database [45]. We then used Genomica [27] to produce a time point-specific TF occupancy network (Figure 3B). This analysis revealed distinct patterns of TF accessing to DNA at different time points. Many known TFs were enriched, such as STAT5, which is an IL-15 downstream signaling molecule and is indispensable throughout the lifetime of NK cells. Deficiency of STAT5a/b has been reported to result in complete elimination of NK cells, demonstrating the important and non-redundant effects of STAT5 [43]. The JAK-STAT pathway has also been shown to be an important signaling pathway used by various cytokines and growth factors [46]. The interferon regulatory factor (IRF) family regulates a variety of processes, including hematopoietic development, tumorigenesis, host immunization, and pathogen defense [47], [48]. IRF2 is required to maintain the normal differentiation of NK cells in a cell-intrinsic manner [49], [50], and IRF2-deficient NK cells show reduced levels of maturation markers and IFN-γ production during stimulation [49], [50]. T-bet and EOMES are members of the T-box family and are known to control different aspects of NK cell differentiation and maturation [21], [51], [52].

In addition, several novel TFs were also enriched, such as FOSL2 and EGR2, suggeting that they are potential regulators of NK cell differentiation. These TFs were assessed along with the other enriched TF families from the motif analysis to identify which family members were expressed or differentially expressed during NK cell differentiation to further filter out true regulators. At each time point, we plotted both the expression value (colored from red to green to represent high to low expression in the gene microarray profile) and the motif enrichment score (shown by the circle size representing the –log *P* value of the enrichment) in the same figure (Figure 3C). We observed that known regulators were highly expressed and enriched at different stages, same as FOSL2 and EGR2. Through the integral analyses (Figure 3A–C), we predict FOSL2 and EGR2 as potential regulators.

DNA sequences that are directly occupied by DNA-binding proteins are protected from transposition, and therefore the resulting sequence “footprint” reveals the presence of a DNA-binding protein at each site, analogous to DNase digestion footprints. The TF “footprint” analysis of our ATAC-seq profile provided further evidence of direct occupancy of a TF candidate on genomic DNA and thus refined the prediction of potential regulators. We illustrated the “footprints” of two known regulators, STAT5 and T-bet, and observed higher DNA accessibility and deeper “footprints” flanking their motifs in the interim and late stages than those in the early stage during NK cell differentiation (Figure 3D). Similarly, “footprints” of the TFs FOSL2 and EGR2 were also deeper and more accessible at the interim and late stages. These data suggest that not only the binding motifs of these two TFs are enriched at stage-specific peaks, but also these TFs most likely physically bind to these accessible chromatin sites, indicating that they are functional regulators of NK cell differentiation (Figure 3D). Overall, the results from footprint analysis agree with those from the HOMER and Genomica’s motif enrichment analyses.

Genes that are regulated by any TF of interest can be predicted by combining the TF motif and “footprint” analyses. We thus predicted the genes regulated by TFs including RUNX, T-bet, FOSL2, and EGR2, and integrated the gene expression profiles at each time point (Figure S5B; Table S2). We found that genes regulated by each of these TFs were also highly expressed at the interim and late stages and were significantly enriched in GO terms of immune system construction and other related functions. TF ETS proto-oncogene 1 (ETS1) was reported to drive early stages of NK cell differentiation [53], and we found that there was a FOSL2-binding site in a dynamically accessible site on the gene body of *ETS1*, suggesting FOSL2 might regulate NK cell differentiation through *ETS1* (Figure 3E, left panel). TF GATA3 was found to regulate liver-specific NK cells, IFN-γ production, and T-bet expression in mice [54]. Similarly, we found that both FOSL2 and EGR2 bind to the gene body of *GATA3* (Figure 3E, right panel), suggesting that the TFs FOSL2 and EGR2 may also regulate NK cell differentiation through regulation of *GATA3*.

### FOSL2 and EGR2 affect NK cell differentiation

FOSL2 belongs to the Fos gene family, which encodes a leucine zipper protein and forms the AP-1 TF complex by dimerization with the JUN family. Thus, FOS proteins have been suggested as key regulators of transformation, differentiation, and cell proliferation. The GO annotations of *FOSL2* include sequence-specific DNA binding, TF activity, and RNA polymerase II specific DNA binding. A previous report has shown that FOSL2 is constitutively expressed in adult T-cell leukemia (ATL), up-regulates *CCR4* expression, and promotes ATL cell growth, together with JunD proto-oncogene, AP-1 transcription factor subunit (JUND) [55]. *EGR2* encodes a protein that contains three tandem C2H2-type zinc fingers. GO annotations of this gene include ligase activity, sequence-specific DNA binding, and TF activity. Previous reports have shown that EGR2 regulate T-cell and B-cell function in homeostasis and adaptive immune responses by controlling inflammation and promoting antigen receptor signaling [56], [57].

**Figure 4:**
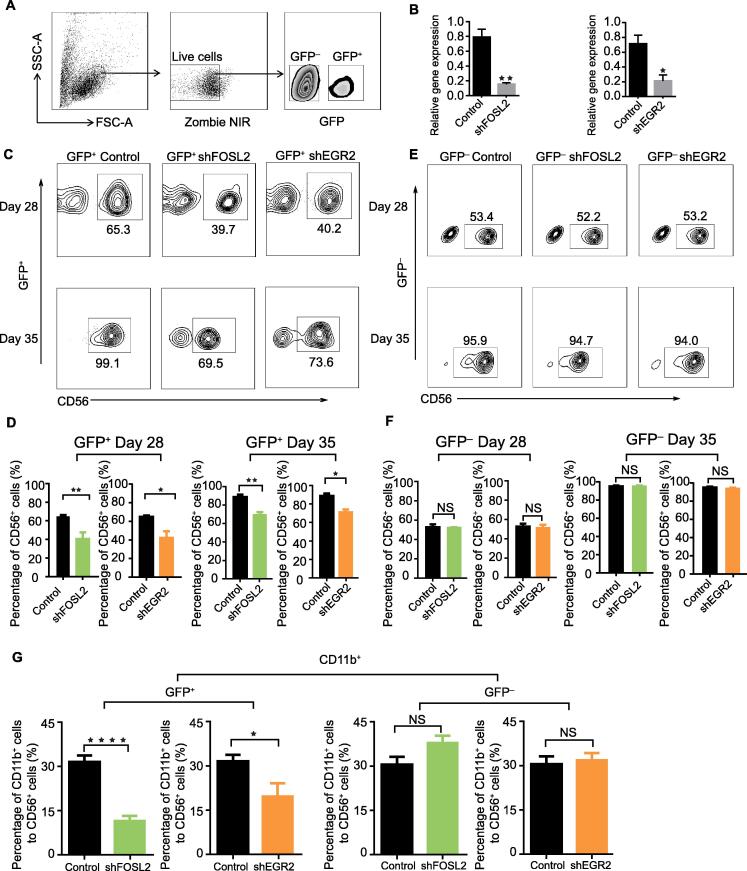
**Function of EGR2 and FOSL2 during NK cell differentiation** **A.** The gating strategy for cultured cells transduced with lentiviruses expressing shFOSL2, shEGR2, or control shRNA via detection of GFP expression. **B.** Knockdown efficiency of *FOSL2* and *EGR2* with shFOSL2 and shEGR2. **C.** Flow cytometric analysis of CD56^+^ cells in the GFP^+^ cells at day 28 (top) and day 35 (bottom). GFP^+^ cells refer to cultured cells successfully transfected with lentiviruses expressing control shRNA (vector with a meaningless fragment), shFOSL2, or shEGR2. **D.** Quantification of CD56^+^ cells in the GFP^+^ cell population at day 28 (left) and day 35 (right). *n* = 5. **E.** Flow cytometric analysis of CD56^+^ in the GFP^−^ cells at day 28 (top) and day 35 (bottom). GFP^−^ cells refer to cultured cells not successfully transfected with lentiviruses expressing control shRNA, shFOSL2, or shEGR2. **F.** Quantification of CD56^+^ cells in the GFP^−^ cell population at day 28 (left) and day 35 (right). *n* = 5. **G.** Quantification of CD11b^+^ cells in the CD56^+^ cell population at day 35 (*n* = 5). Data are presented as the mean ± SEM. *, *P* < 0.05; **, *P* < 0.001; ***, *P* < 0.0005; ****, *P* < 0.0001. *P* values were estimated from Student’s *t*-test. SSC-A, side scatter area; FSC-A, forward scatter area; NIR, near infrared.

Since their regulatory functions in NK cell differentiation have not been well characterized, we performed loss-of-function experiments to assess the effects of *FOSL2* and *EGR2* on NK cell differentiation. We first validated their expression levels with real-time PCR, and found that their expression gradually increased in later stages of NK cell differentiation, consistent with our bioinformatics analysis (Figure S6A). Subsequently, we infected the cultured cells with TF-specific shRNA- and control shRNA-containing lentiviruses, which are represented by GFP expression (Figure 4A). We then sorted the GFP-positive cells and observed dramatically reduced expression levels of FOSL2 and EGR2 (Figure 4B), suggesting positive targeting of the TF-specific shRNAs. During NK cell differentiation, we observed a nearly 30% reduction in the proportion of differentiated NK cells in GFP-positive cells at day 28 and day 35 (Figure 4C and D). However, in the non-successfully transfected (GFP-negative) cells, the proportion of differentiated NK cells was not affected at the same time point (Figure 4E and F). These results indicate that knockdown of *FOSL2* and *EGR2* expression, but not viral infection, inhibits NK cell differentiation, suggesting that FOSL2 and EGR2 are necessary for NK cell differentiation. We then tested another important marker of NK cell maturation CD11b [1], and found that it was significantly reduced in GFP-positive NK cells, suggesting that FOSL2 and EGR2 might affect NK cell maturation (Figure 4G). Overall, we predicted these two TFs FOSL2 and EGR2 as key regulators based on ATAC-seq and gene microarray profile analyses, and then experimentally verified that they indeed affect NK cell differentiation.

In addition, we attempted to identify the signaling pathways through which FOSL2 and EGR2 were involved in driving NK cell differentiation by performing module map analysis of all the differential peaks. Eighteen modules were identified according to the patterns of their accessibility (Table S3), several of which were significantly enriched with known TFs that regulate NK cell differentiation (Figure S6B), including *FOSL2* and *EGR2*, and several other genes from the JAK-STAT pathway, such as the *STAT* family, *IRF2*, *TBX21*, and *GATA3*. These results suggested that FOSL2 and EGR2 may regulate NK cell differentiation through the JAK-STAT pathway (Figure S6C).

### Transcriptional regulatory network dynamics during NK cell differentiation

**Figure 5:**
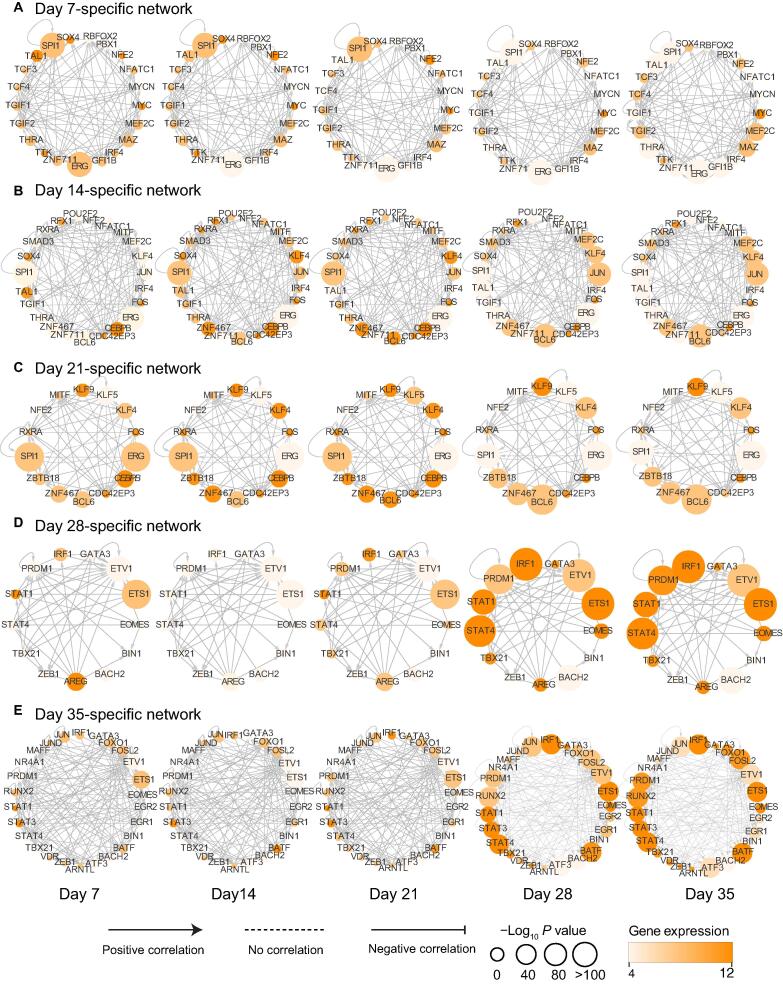
**Transcriptional regulatory network during NK cell differentiation** **A.**–**E.***Cis*-regulatory networks between TFs (nodes) enriched in ≥1 gene set and specifically expressed (FC > 1.5) at day 7 (A), day 14 (B), day 21 (C), day 28 (D), and day 35 (E). Nodes represent TFs with gene expression levels and TF enrichment scores at day 7, day 14, day 21, day 28, and day 35 (from left to right). The arrow at the edge from node A to node B indicates that TF A regulates TF B by binding to the promoter region of TF B. The color of each node indicates the expression level of the gene encoding that TF, and the size of the circle represents the significance of the motif enrichment according to the *P* value. The types of edges indicate the Pearson correlation between the gene expression profiles of the connected TFs: positively correlated (PCC > 0.4); negatively correlated (PCC < −0.4); no correlation (−0.4 ≤ PCC ≤ 0.4, dashed line). FC, fold change; PCC, Pearson correlation coefficient.

The dramatic chromatin accessibility differences during NK cell differentiation prompted us to check the time point-specific transcriptional regulatory network. Although some TFs have been discovered to regulate NK cell differentiation, little is known about the dynamics of the entire regulatory network during this process. Since the TF footprint pattern from the ATAC-seq reads could simultaneously and directly reveal the binding profiles of hundreds of TFs with known cognate motifs [22], together with gene expression profiles, we could construct a regulatory network at each time point and assess how it changes during NK cell differentiation. First, we used HOMER to identify enriched TFs that bind to the cluster I and cluster II peaks shown in Figure 3A (*P* < 0.05). We then examined the gene expression profiles of these TFs and found that 120 TFs were expressed at least one stage during the differentiated process. By applying differential analysis, we obtained 14–26 TFs that were distinctly expressed at each stage with fold change (FC) > 1.5 (Figure S7), and defined them as the nodes of the regulatory network. The connections (edges) between any two TFs were defined as follows: if the TF A’s motif is on the promoter of TF B, then we say TF A regulates TF B and draw an arrow from TF A to TF B. Here, only TFs that were expressed at the specific time point were considered [58]. Using this method, we constructed the transcriptional regulatory network at each time point with both the enrichment (*P* value) and expression information for all the relevant TFs (Figure 5A–E). Interestingly, intensive interconnections appeared for day 7-specific TFs, and quickly vanished after two weeks (Figure 5A). In contrast, the day 35-specific network gradually grew through the induction of relevant TFs. Many known regulators, such as EOMES, TBX21, ETS1, PR/SET domain 1 (PRDM1), and GATA3, as well as FOLS2 and EGR2, were increasingly enriched in the network (Figure 5E). Similar phenomena were also observed on other networks (Figure 5B–D). The dynamics of the transcriptional regulatory networks seem to explain the increase in the proportion and the differentiation of NK cells.

## Discussion

NK cells are important innate immune cells that have been recognized as efficient effector cells to treat tumors. To better understand the differentiation machinery of NK cells and to identify new regulators in the process, we studied the landscape of active elements during NK cell differentiation using the sensitive ATAC-seq method. As a result, we find three distinct clusters of DNA accessible elements. Further analysis shows that the chromatin accessibility is correlated well with the expression levels of the corresponding genes. In short, this study provides an epigenomic landscape and dynamics of NK cell differentiation and presents fundamental profiles for studying the relationship between chromatin accessibility, gene expression, and cell growth during this process (Figure 2).

TFs bind to their motifs and are often obligate nucleosome evictors, and therefore we can use ATAC-seq to predict critical regulators in NK cell differentiation [26]. By motif analysis in HOMER, we find that several known TFs are enriched at different stages during NK cell differentiation. Similar results have also been obtained using Genomica (Figure 3B). The discovery of known regulators strongly supports the reliability of our analysis. Furthermore, by integrating results from HOMER, Genomica, and motif footprint analysis, we have identified two novel TFs, FOSL2 and EGR2, which are essential for NK cell maturation. Knockdown of either of these two TFs significantly inhibits NK cell transformation in the *in vitro* NK cell differentiation system. Module map analysis suggests that these two TFs may regulate NK cell differentiation through the JAK-STAT pathway, and therefore further studies of this pathway may facilitate the generation of NK cells and thus promote the NK cell-based immunotherapy. Overall, this study also provides a framework to identify new regulators from chromatin accessible data for NK cell differentiation.

TFs do not usually function alone, they always interact with other molecules to fulfill their unique roles. Hence, we have depicted the transcriptional regulatory networks at different stages during NK cell differentiation. In order to construct a stable transcriptional regulatory network, we have performed a rigorous screening of TFs to avoid stochastic fluctuations, and integrated both the enrichment (*P* value) and expression information for all the relevant TFs. Therefore, although alterations in either the enrichment or gene expression cutoff may result in different networks, the most critical TFs to the regulatory process still remain. However, since the screening of TFs mainly rely on the gene microarray, which is not as accurate as RNA-seq, the structure of these predicted networks may not be very robust.

From our previous study, we notice that with a minimal cytokine cocktail, we can generate sufficient number of functional NK cells that express the cytokines necessary for NK cells and have a high effect on tumor cells [19]. Although there may also be a certain proportion of other lymphocytes, *in vitro* expansion of NK cells from peripheral blood (PB) or UCB cells has been successfully performed and developed for several clinical strategies to treat cancers [4], [5], [59], [60], [61]. Therefore, a comprehensive understanding of the regulatory patterns at each differentiation stage of the *in vitro*-derived NK cells in this system will help to uncover the underlying mechanisms of NK cell differentiation. The transcriptional regulatory networks revealed in this study will lay the foundation for efficient *in vitro* production of effective NK cells, thus facilitating NK cell-based immunotherapy. Moreover, we have identified two novel TFs, FOSL2 and EGR2, as essential regulators in controlling NK cell maturation and function. We have also predicted the potential signaling pathways in which these two TFs may be involved and have illustrated the dynamics of the transcriptional regulatory networks during NK cell differentiation. In spite of the advantages of our strategy, there are two main limitations of this study. 1) Although induced NK cells are very similar to those produced *in vivo*, these two types of NK cells are not identical. We have observed certain differences between the induced NK cells and the NK cells produced *in vivo* in terms of chromatin accessibility (data not shown). 2) Before NK cells are fully developed, there is always a mixture of cell populations with stem cells, NKP cells, iNK cells, mature NK cells, and other cell types that we are unable to delineate at this moment, since any bulk cell-based analysis may neglect the huge heterogeneity between cells by default. Therefore, to fully uncover the regulatory mechanism, single cell technologies are required in the future to further delineate the cell-to-cell heterogeneity and regulatory dynamics at the single cell precision.

## Materials and methods

### Samples

UCB samples were collected at birth from women with normal, full-term deliveries at Anhui Provincial Hospital, Hefei, China, after receiving their written informed consent. The culture procedure for NK cell differentiation from UCB CD34^+^ cells has been previously described [19].

### Immunofluorescence staining

The cultured cells were post-fixed in 4% paraformaldehyde, blocked with 10% goat serum (Catalog No. 31872; ThermoFisher Scientific, Grand Island, NY), before incubating with primary antibodies at 4 °C overnight. Secondary antibodies were then added and incubated at 37 °C for 1 h after washes. Cell nuclei were stained with DAPI for 5 min at room temperature. Confocal images were acquired using a Zeiss LS710 microscope.

### RNA isolation and real-time PCR

Cultured cells were lysed in TRIzol reagent (Catalog No. 15596026; ThermoFisher Scientific), and total RNA was extracted following the manufacturer’s instructions. cDNA was synthesized with Moloney murine leukemia virus reverse transcriptase (Catalog No. 28025013; ThermoFisher Scientific) and oligo (dT) 20 primers. Then, SYBR Premix Ex Taq (Catalog No. RR420L; TaKaRa, Dalian, China) was used for real-time PCR using primers with sequences listed in Table S4. The data were analyzed using the 2^−^^ΔΔCt^ method.

### Lentivirus production and transduction

To produce lentiviral particles, 293 T cells were transfected with the plasmids PLKO.1, pRRE, pREV, or pVSV-G using Lipofectamine 2000 (Catalog No. 11668-019; Invitrogen, Grand Island, NY) following the manufacturer’s protocol. Then, we harvested the supernatants 48 h and 72 h post-transfection. To remove cell debris, supernatants were centrifuged at 3000 rpm for 10 min, and then, the lentivirus particles were concentrated by ultracentrifugation at 50,000 *g* for 2 h at 4 °C. Finally, the virus particles were gently resuspended in Hanks' balanced salt solution (HBSS) and stored at −80 °C. After UCB CD34^+^ cells were cultured with multiple cytokines for 14–18 days, we incubated the lentiviruses and the cultured cells with polybrene (Catalog No. H9268-10G; Sigma Aldrich, Shanghai, China) (5 µg/ml) for 30 min and the mixture was subjected to centrifugation at 1000 rpm for 70 min at 10 °C.

### Statistical analysis

Statistical significance was analyzed using unpaired two-tailed Student's *t*-test or one-way ANOVA. *P* values less than 0.05 were considered statistically significant.

### ATAC-seq and analysis

ATAC-seq was performed as previously described [22], and 2 × 150 paired-end sequencing was performed on an Illumina HiSeq X-10 to yield, on average, 78 M reads/sample. Sample reads from biological replicates were then grouped together and divided into eight categories: day 7, day 14, day 19, day 21, day 24, day 26, day 28, and day 35. Intrinsic analysis and other ATAC-seq analysis was performed same as our previous work (see File S1) [26], [62].

### Differential analysis

All samples were grouped into eight categories (16 samples): day 7, day 14, day 19, day 21, day 24, day 26, day 28, and day 35. Data normalization and significance analysis were performed via pairwise comparison between the eight categories using DESeq2 [63] with *P* < 0.01, log_2_ FC > 5, and FDR < 0.01, and intrinsic analysis [26] with a z-score > 1. We finally obtained 6401 differential peaks. Unsupervised clustering was performed using Cluster 3.0 and visualized in Treeview. GREAT [33] was used to predict enriched functions and GO terms.

### Identification of stage-specific peaks

As shown in Figure S3, each stage (*e.g.*, day 7) consists of genes that were more highly expressed (FC > 1.5) at this stage compared with all the other stages, and we defined these genes as stage-specific genes. Similarly, we defined peaks that were more accessible (FC > 1.5) in one stage compare with all the other stages as stage-specific peaks. Peaks that were accessible at all stages were defined as conservative peaks.

### TF motif enrichment analysis

HOMER [29] was used to perform the TF enrichment analysis with the following options: findmotifs.pl input.fa fasta output/ (Figure 3A) and findmotifs.pl input.fa human uotputdir -fasta bg.fa (Figure S5A). TF enrichment analysis was performed in Genomica. TF “footprint” analysis was performed in the same way as described in our previous work [27].

### Gene module and STRING analysis

Gene module analyses were performed using WGCNA [64] with the options SoftPower = 20, minModuleSize = 30. Protein–protein interaction analyses were performed using STRING [65].

### Construction of transcriptional regulatory networks

We used HOMER to find the TFs that bind to cluster I and cluster II peaks and obtained TFs that could regulate stage differentially expressed elements (*P* < 0.05). We define the region 2 kb upstream of the transcription start site as a promoter. If TF A bound to the promoter of TF B, we defined TF A as a regulator of TF B and then constructed a transcriptional regulatory network. Networks of TFs were assembled from TFs that were expressed in at least one sample. An edge between TF A and TF B indicated that TF A binds to the promoter of TF B [58].

## Ethical statement

This study was approved by the Ethics Committee of the University of Science and Technology of China.

## Data availability

The raw ATAC-seq data have been deposited in the Genome Sequence Archive [66] at the National Genomics Data Center, Beijing Institute of Genomics, Chinese Academy of Sciences / China National Center for Bioinformation (GSA: CRA000846) and are publicly accessible at http://bigd.big.ac.cn/gsa.

## CRediT author statement

**Kun Li:** Conceptualization, Methodology, Software, Writing - original draft, Visualization. **Yang Wu:** Conceptualization, Data curation, Validation, Writing - original draft. **Young Li:** Data curation, Validation. **Qiaoni Yu:** Methodology. **Zhigang Tian:** Supervision. **Haiming Wei:** Conceptualization, Writing - review & editing. **Kun Qu:** Conceptualization, Writing - review & editing, Visualization, Project administration, Funding acquisition. All authors read and approved the final manuscript.

## Competing interests

All authors have no competing financial interests.

## Supplementary material

Supplementary File S1 **Supplementary methods**.

Supplementary Figure S1 **NK cell number and ATAC-seq data quality**. **A.** Fractions of CD56^+^CD3^-^ cells in the total gated cells during a 35-day time course. **B.** and **C.** Quality control analysis of ATAC-seq data. TSS enrichment score for all samples is shown in panel B, and the fragment length distribution of all the mapped reads is shown in panel C**. D.** Normalized ATAC-seq profiles of known genes including *CD34*, *KIT* (CD117), *KLRD1* (CD94), and *NCAM1* (CD56) gene loci at different stages during NK cell differentiation. **E.** Normalized ATAC-seq profiles of known TF genes including *EOMES* (left) and *GATA3* (right) gene loci at different stages during NK cell differentiation. ATAC-seq signals were obtained from the UCSC Genome Browser. TSS, transcription start site.

Supplementary Figure S2 **Correlation analysis on samples during NK cell development**. **A.** Correlation analysis on the replicates at each time point. *r* at the top indicates PCC value. **B.** Heatmap of the Pearson correlation coefficient between all the samples with unsupervised clustering performed in Cluster 3.0. PCC, Pearson correlation coefficient.

Supplementary Figure S3 **Changes in DNA open sites during NK cell development**. **A.** Venn diagram of peaks identified at each stage of NK cell differentiation. Stage-specific peaks are defined as the peaks that were identified only at a specific time point, whereas conserved peaks refer to the peaks that were identified at all stages during the process. **B.** The top most significant GO terms of all the stage-specific peaks (green) and conserved peaks (orange).

Supplementary Figure S4 **Different stages of NK cell development have specific regulatory elements. A.** Heatmap of all the 6401 differential regulatory elements in all the samples. Each column is a sample, and each row is a peak. Samples and peaks were organized by two-dimensional unsupervised hierarchical clustering. The color scale shows the relative ATAC-seq signal intensities as indicated. Top: samples at all time points were categorized into three groups, early stage: day 7–21 (orange); interim stage: day 24–28 (yellow) and late stage: day 35 (green). Samples from the same cluster are labeled with the same color. Right: differential peaks are categorized into three clusters. **B.** Distance from all the peaks in cluster I, II, and III to their nearest genes. Known TFs regulating NK cell differentiation are labeled. C. PCA of chromatin accessibility during NK cell differentiation. Three clusters were identified: early (day 7–21), interim (day 24–28), and late (day 35). PCA, principle component analysis.

Supplementary Figure S5 **TFs enriched at different stages of NK cell development**. **A.** The top 10 TF motifs enriched in cluster I (left) and cluster II (right) peaks, with enrichment *P* values estimated from HOMER. TFs known to regulate NK cell differentiation are color-coded. **B.** Gene expression and enriched GO terms of TF-binding genes. Unsupervised hierarchical clustering was performed. Expression heatmap of the genes predicted to be regulated by the same TF (RUNX, T-bet, FOSL2, or EGR2) is shown on the left, whereas the top 10 most significant GO terms enriched in up-regulated (orange) and down-regulated (green) genes predicted to be regulated by each TF are shown on the left.

Supplementary Figure S6 **Signaling pathways involving FOSL2 and EGR2**. **A.** Real-time qPCR analysis (*n* = 3) of the genes *FOSL2* (left) and *EGR2* (right). Data from three replicates are presented as mean ± SEM. **B**. Module map analysis in Genomica: representative modules from module map analysis in Genomica. For each module, the most significantly enriched TF in the module is shown at the top, and the chromatin accessibility changes of the peaks in the corresponding module compared with day 7 are shown at the bottom. Genes associated with peaks in the respective module are listed on the right. **C**. Signaling pathways of the known (green circles) and predicted (olivine circles) TFs that regulate NK cell differentiation.

Supplementary Figure S7 **Define time-specific TFs based on differential expression analysis**. **A.** FC of expression distribution of all TFs at a specific day *vs*. other time points during NK cell differentiation. FC of 1.5 indicates 2× STD away. Time points are color coded. **B.** Number of TFs at different FC cutoffs. **C**. The ratio of known TFs at different FC cutoffs versus all known TFs. Known TFs here refer to TFs regulating NK cell development reported in the literature. FC, fold change; STD, standard deviation.
