# Supplementary material for: Landscape and Dynamics of the Transcriptional Regulatory Network During Natural Killer Cell Differentiation
Source: Genomics Proteomics Bioinformatics. 2020 Dec 30;18(5):501–15. doi: 10.1016/j.gpb.2020.12.003 (PMC8377244; doi:10.1016/j.gpb.2020.12.003)
Supplement: Supplementary File S1 — Supplementary methods. [file mmc1.docx]

**File S1 Supplementary methods**

**Methods**

**Generation of NK cells differentiated from UCB CD34^+^ cells**

The culture procedure for NK cell differentiation from UCB CD34-positive cells has been previously described [1].

**Flow cytometry**

The cell suspensions were labeled with ﬂuorochrome-conjugated monoclonal antibodies to analyze the expression levels of cell membrane and intracellular molecules. They were detected using an LSR II flow cytometer (BD Biosciences) and analyzed with FlowJo software (Tree Star). The anti-CD56 (Catalog No. 362546), anti-Pu.1 (Catalog No. 658009), anti-GATA3 antibodies (Catalog No.653811), and Zombie NIR Fixable Viability kit (Catalog No.423105) were obtained from Biolegend (San Diego, CA). Anti-CD3 (Catalog No557749), anti-CD11b (Catalog No.550019), and anti-IFN-γ (Catalog No.559327) antibodies were from BD Pharmingen (San Jose, CA), as well as anti-T-bet antibody (Catalog No.12-5825-82) was from eBioscience (Grand Island, NY).

**Lentiviral vector construction**

Short hairpin (sh) RNA sequences targeting *FOSL2* and *EGR2* (Table S4) and a negative control sequence were synthesized by Sangon Biotech and then cloned into the lentivirus shRNA expression vector pLKO.1 (Addgene).

**Primary data processing and peak calling**

Adapter sequences were trimmed from the raw reads using the adapter TrimmingModified [2], and then, the trimmed reads were mapped to Hg19 using Bowtie2 [3] (with the option --very-sensitive). PCR duplicates were removed as described [4], and uniquely mapped reads were shifted +4/-5 bp according to the strand of the read, so that the first base of each read represented the Tn5 cleavage position. All mapped reads were then extended to 50 bp centered through the cleavage [5] position. Reads mapped to repeated regions and chromosome M were removed. Peak calling was performed using MACS2 [6] with the options - f BED -g hs, -q 0.01, --nomodel, and --shift 0. Peaks in each sample were called out for quality control purposes. Sample reads from biological replicates were then grouped together and divided into 8 categories: day 7, day 14, day 19, day 21, day 24, day 26, day 28, and day 35. Reads from the same category were concatenated, and then, peak calling was performed using the same options described above. Peaks from all the categories were then merged together into a unique peak list, and the numbers of raw reads mapped to each peak at each condition were quantified using the intersectBed function in BedTools [7]. Raw counts in peaks were normalized using the DESeq package in R [8]. Peak intensity was defined as the log2 of the normalized counts. After these steps, an N×M data matrix was obtained, in which N indicates the number of merged peaks, M indicates the number of samples, and D_i, j_ indicates the intensity of the peak i (I = 1 to N) in sample j (j = 1 to M). For each pair of samples, Pearson correlations were calculated based on the log2 normalized counts of all the peaks. Unsupervised clustering of the Pearson correlation matrix was performed using Cluster 3.0 [9] and visualized in Java Treeview.

**Data quality control**

ATAC-seq data quality control was comprehensively studied in our previous work [10].The ability ATAC-seq to detect accessible sites was validated by comparing the ATAC-seq signal with the results of DNaseI hypersensitivity sequencing (DHS-seq), which serves as the gold standard assay and positive control for open chromatin detection. We found that ATAC-seq was highly reproducible between replicates. From an irreproducibility discovery rate (IDR) analysis [11], which is an ENCODE-specified method to evaluate data reproducibility across replicates, we found that the number of reproducible peaks plateaued between 11–12 million mappable reads, irrespective of the IDR cutoff [10]. In this study, we obtained, on average, 11.3 million uniquely mapped reads, suggesting that the sequencing in this study was sufficiently deep to confidently capture the majority of regions of interest. We then used several metrics to evaluate the quality of each sample, including the number of raw reads, overall alignment rate, final mapped reads, final mapped rate, percentage of reads mapped to chrM, percentage of reads mapped to repeat regions (black list), percentage of reads filtered out by low MAPQ scores, percentage of PCR duplicates, TSS enrichment score (reads enriched at +/-2 kb around the TSS versus the background), read length distribution and number of peaks. We finally filtered out 16 high-quality samples.

**Significance analysis**

Samples were then grouped into 8 categories (16 samples): day 7, day 14, day 19, day 21, day 24, day 26, day 28, and day 35. Data normalization and significance analysis were performed via pairwise comparison between the 8 categories using DESeq2 [8], with a P < 0.01, FDR < 0.01, log2-fold change > 5, and intrinsic analysis [10] and with a z-score > 1. We finally obtained 6401 differential accessible peaks. Unsupervised clustering was performed using Cluster 3.0 and visualized in Treeview. The ATAC-seq signal in each category was normalized to its corresponding sequencing depth. Gene ontology and other enriched functions of cis-regulatory regions were predicted using GREAT [12].

**Intrinsic analysis**

We defined a log2-fold change in peak i as:

log2fdc_i_ = max (D_i,j = 1,M_ ) – min(D_i,j = 1,M_ ).

We defined a correlation matrix C where C_p, q_ is the Pearson correlation between samples p and q in which all the peaks were included. Similarly, we defined a correlation matrix C^i^ where C^i^_p, q_ is the Pearson correlation between samples p and q in which all peaks but peak i were included. We defined a delta matrix as deltaC^i^ = C – C^i^. We then defined a

wbScore_i_ = average (deltaC^i^ _replicates_) – average (deltaC^i^ _non − replicates_).

Replicates were defined as samples obtained from the same donor at the same time, and non-replicates as otherwise. For peak i, a greater wbScore_i_ is associated with less variance of the peak intensity within replicates and greater variance between non-replicates. We then calculated the average and standard deviation of all wbScores (from I = 1 to N).

We performed a permutation analysis that randomly ranked the samples and assigned replicates 1000 times, and we estimated a false discovery rate FDR_i = 1,N_ for each accessible element as the frequency of seeing a wbScore_i_ obtained from a random ranking greater than the observed wbScore_i_ from an as is ordering.

We defined peak i as significantly differentially accessible if

wbScore_i_ > average(wbScore_i = 1,N_) + sd(wbScore_i = 1,N_).

Therefore, we finally obtained 6401 differential accessible peaks using the following options: P < 0.01, FDR < 0.01, log_2_-fold change > 5 and z-score > 1.

**Stage-specific TFs**

In Figure S7 each stage (*e.g.*, day 7) consists of TFs that were induced (> 1.5-fold change) at that stage compared with all the other stages. We believe that the TF is significantly enriched at this stage. Percent of known TFs indicates the ratio of known TFs retained at all stages to all known TFs.

**TF enrichment analysis using Genomica**

The input motif set was obtained from JASPAR (http://jaspar.genereg.net/) on vertebrates. We searched for input motifs in each significant differential peak using HOMER [13] and generated a peak versus motif matrix, where each row is a peak and each column is a motif. A value of 1 in the matrix indicates that the motif is found in the peak, and value 0 represents NOT. Based on the peak significance analysis, we obtained a peak-by-sample matrix in which each row is a peak and each column is a sample, and values in the matrix represent the peak intensity in the corresponding sample. We then mean-centered the values in each peak to obtain the relative fold changes of each peak across all the samples. By integrating these two matrices into Genomica [10] using the ModuleMap algorithm, we were able to ask whether and to what extent a motif was enriched in each sample, and obtain a motif by sample matrix, in which each row is a motif, each column is a sample, and the values in the matrix represent the significance of enrichment by –log(*P* value). If the average fold change of a motif in a sample was positive, then the motif was defined as positively enriched in the sample; if the average fold change of a motif in a sample was negative, then the motif was defined as negatively enriched in the sample. The top enriched/depleted motifs between different samples was defined as the –log(*P* value) difference between normal samples and disease samples (herein different time point samples). The higher this value, the more enriched this motif was in normal samples; the lower this value, the more enriched this motif was in disease samples.

**TF “footprint” analysis**

The genome-wide motif footprint analysis was performed using PIQ v1.2 [14]. For footprint, we adjusted the read start sites to represent the center of the transposon binding event (see above). Previous descriptions of the Tn5 transposase have shown that the transposon binds as a dimer and inserts two adaptors separated by 9 bp [15]. Therefore, we modified the read aligned file in sam format by offsetting +4 bp for all the reads aligned to the forward strand and -5 bp for all the reads aligned to the reverse strand. We then converted a shifted base sam file to bam format and sorted the bam file using samtools. PIQ predicted genomic occupation of 242 motifs with binding affinity estimated by purity scores. We filtered the PIQ predictions using a purity score cutoff at 0.7 and overlaid these predictions with reads in each category. We then averaged the overlaid read counts of a 150 bp genomic region centered by the motif sites for motif footprints.

**References**

[1] Wu Y, Li Y, Fu B, Jin L, Zheng X, Zhang A, et al. Programmed differentiated natural killer cells kill leukemia cells by engaging SLAM family receptors. Oncotarget 2017;8:57024–38.

[2] Xu J, Carter AC, Gendrel AV, Attia M, Loftus J, Greenleaf WJ, et al. Landscape of monoallelic DNA accessibility in mouse embryonic stem cells and neural progenitor cells. Nat Genet 2017;49:377–86.

[3] Langmead B, Salzberg SL. Fast gapped-read alignment with bowtie 2. Nat Methods 2012;9:357–9.

[4] Buenrostro JD, Giresi PG, Zaba LC, Chang HY, Greenleaf WJ. Transposition of native chromatin for fast and sensitive epigenomic profiling of open chromatin, DNA-binding proteins and nucleosome position. Nat Methods 2013;10:1213–8.

[5] Chang LW, Payton JE, Yuan W, Ley TJ, Nagarajan R, Stormo GD. Computational identification of the normal and perturbed genetic networks involved in myeloid differentiation and acute promyelocytic leukemia. Genome Biol 2008;9:R38.

[6] Zhang Y, Liu T, Meyer CA, Eeckhoute J, Johnson DS, Bernstein BE, et al. Model-based Analysis of ChIP-Seq (MACS). Genome Biol 2008;9:R137.

[7] Quinlan AR, Hall IM. BEDTools: a flexible suite of utilities for comparing genomic features. Bioinformatics 2010;26:841–2.

[8] Anders S, Huber W. Differential expression analysis for sequence count data. Genome Biol 2010;11:R106.

[9] de Hoon MJL, Imoto S, Nolan J, Miyano S. Open source clustering software. Bioinformatics 2004;20:1453–4.

[10] Qu K, Zaba LC, Giresi PG, Li R, Longmire M, Kim YH, et al. Individuality and variation of personal regulomes in primary human T cells. Cell Syst 2015;1:51–61.

[11] Li Q, Brown JB, Huang H, Bickel PJ. Measuring reproducibility of high-throughput experiments. Ann Appl 2011;5:1752–79.

[12] McLean CY, Bristor D, Hiller M, Clarke SL, Schaar BT, Lowe CB, et al. GREAT improves functional interpretation of cis-regulatory regions. Nat Biotechnol 2010;28:495–U155.

[13] Heinz S, Benner C, Spann N, Bertolino E, Lin YC, Laslo P, et al. Simple combinations of lineage-determining transcription factors prime cis-regulatory elements required for macrophage and B cell Identities. Mol Cell 2010;38:576–89.

[14] Sherwood RI, Hashimoto T, O'Donnell CW, Lewis S, Barkal AA, van Hoff JP, et al. Discovery of directional and nondirectional pioneer transcription factors by modeling DNase profile magnitude and shape. Nat Biotechnol 2014;32:171–8.

[15] Adey A, Morrison HG, Asan, Xun X, Kitzman JO, Turner EH, et al. Rapid, low-input, low-bias construction of shotgun fragment libraries by high-density in vitro transposition. Genome Biol 2010;11:R119.
