# Supplementary material for: Landscape and Dynamics of the Transcriptional Regulatory Network During Natural Killer Cell Differentiation
Source: Genomics Proteomics Bioinformatics. 2020 Dec 30;18(5):501–15. doi: 10.1016/j.gpb.2020.12.003 (PMC8377244; doi:10.1016/j.gpb.2020.12.003)
Supplement: Supplementary Figure S1 — NK cell number and ATAC-seq data quality. A. Fractions of CD56+CD3- cells in the total gated cells during a 35-day time course. B. and C. Quality control analysis of ATAC-seq data. TSS enrichment score for all samples is shown in panel B, and the fragment length distribution of all the mapped reads is shown in panel C. D. Normalized ATAC-seq profiles of known genes including CD34, KIT (CD117), KLRD1 (CD94), and NCAM1 (CD56) gene loci at different stages during NK cell differentiation. E. Normalized ATAC-seq profiles of known TF genes including EOMES (left) and GATA3 (right) gene loci at different stages during NK cell differentiation. ATAC-seq signals were obtained from the UCSC Genome Browser. TSS, transcription start site. [file mmc2.pdf]

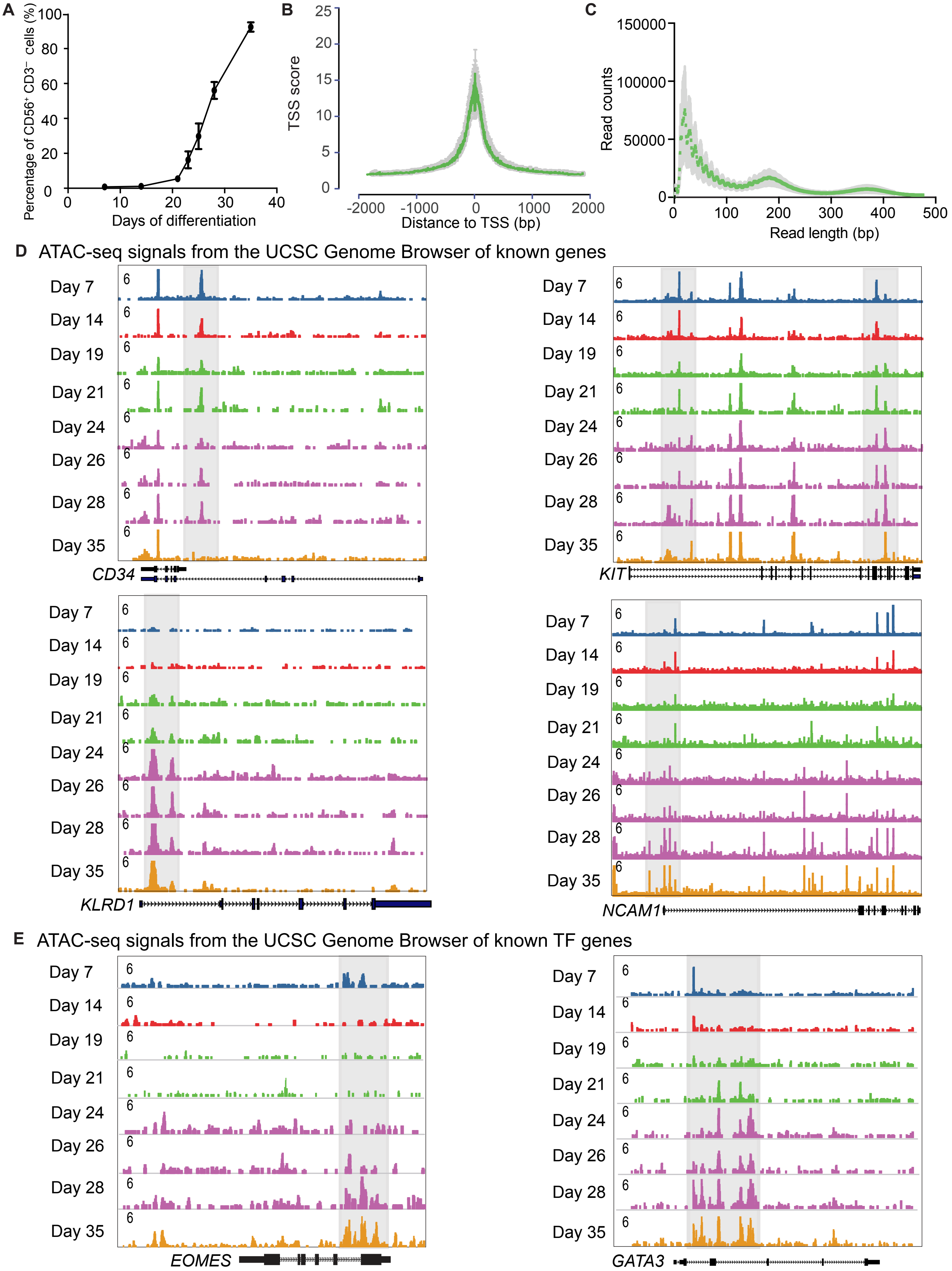
A
Percentage of CD56⁺ CD3⁻ cells (%)
Days of differentiation
B
TSS score
Distance to TSS (bp)
C
Read counts
Read length (bp)
D ATAC-seq signals from the UCSC Genome Browser of known genes
Day 7
Day 14
Day 19
Day 21
Day 24
Day 26
Day 28
Day 35
CD34
KIT
KLRD1
NCAM1
E ATAC-seq signals from the UCSC Genome Browser of known TF genes
EOMES
GATA3
