# Supplementary figures and images for: Landscape and Dynamics of the Transcriptional Regulatory Network During Natural Killer Cell Differentiation
Source: Genomics Proteomics Bioinformatics. 2020 Dec 30;18(5):501–15. doi: 10.1016/j.gpb.2020.12.003 (PMC8377244; doi:10.1016/j.gpb.2020.12.003)

**A**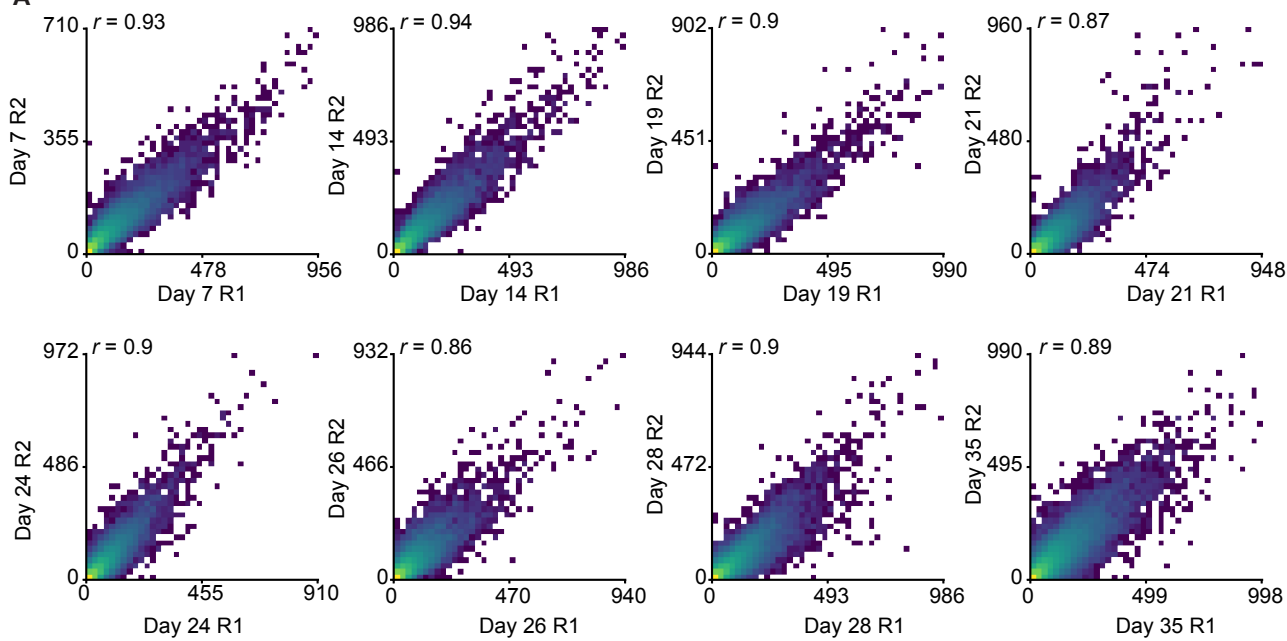**B**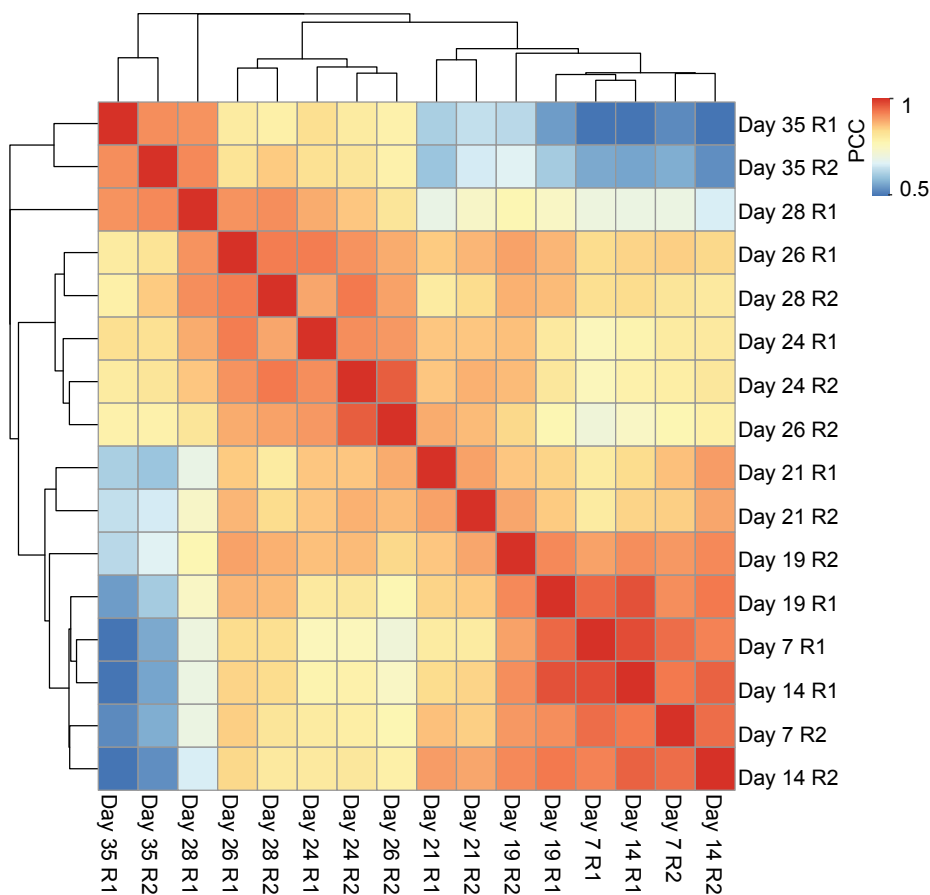

Supplement: Supplementary Figure S2 — Correlation analysis on samples during NK cell development. A. Correlation analysis on the replicates at each time point. r at the top indicates PCC value. B. Heatmap of the Pearson correlation coefficient between all the samples with unsupervised clustering performed in Cluster 3.0. PCC, Pearson correlation coefficient. [file mmc3.pdf]

A

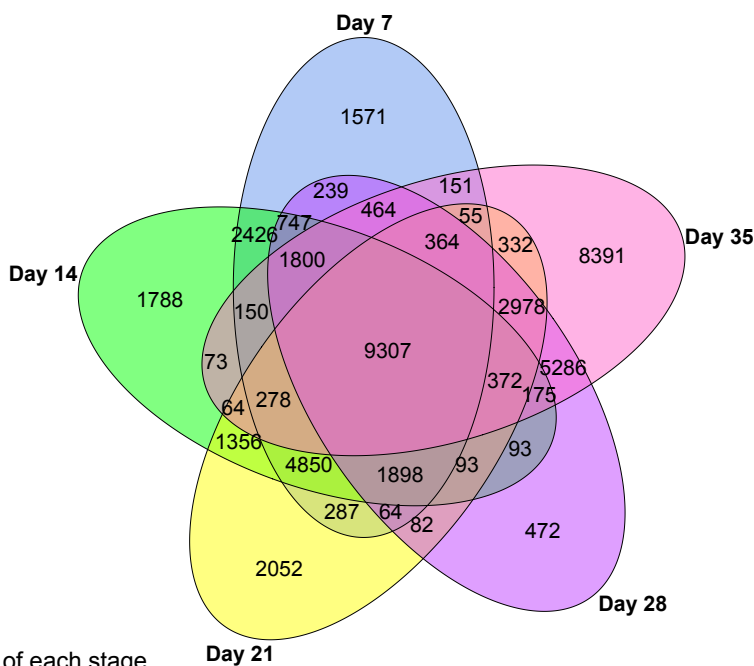

## B Significant GO terms of each stage

Day 7

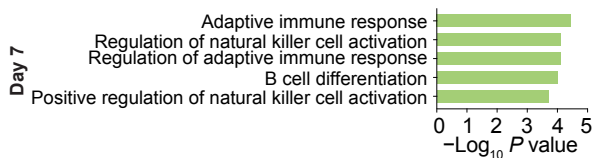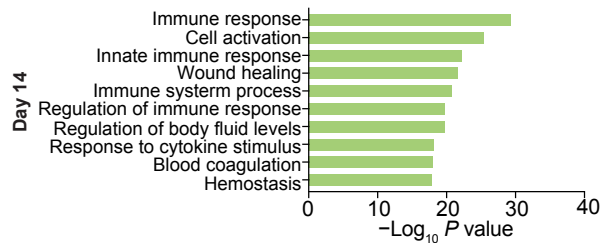

Day 21

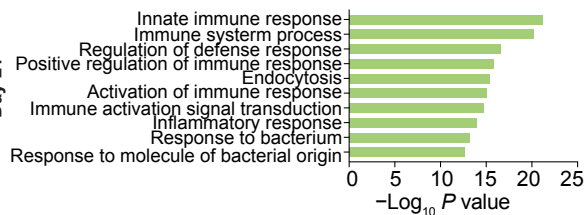

Day 28

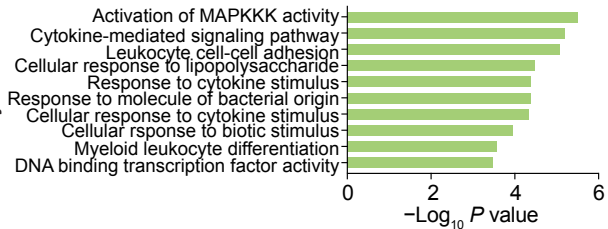

Day 35

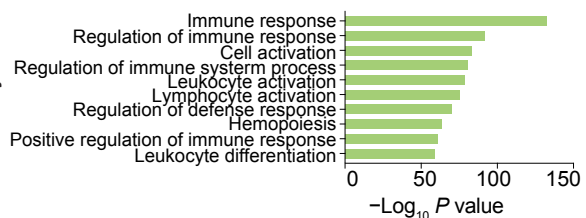

Conserved peaks

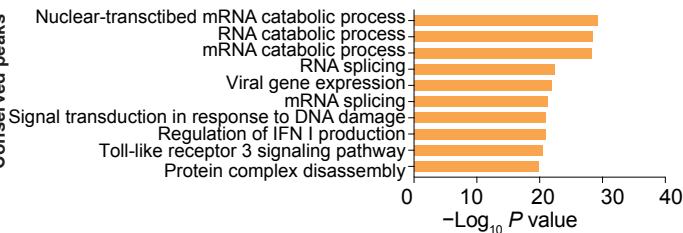

Supplement: Supplementary Figure S3 — Changes in DNA open sites during NK cell development. A. Venn diagram of peaks identified at each stage of NK cell differentiation. Stage-specific peaks are defined as the peaks that were identified only at a specific time point, whereas conserved peaks refer to the peaks that were identified at all stages during the process. B. The top most significant GO terms of all the stage-specific peaks (green) and conserved peaks (orange). [file mmc4.pdf]

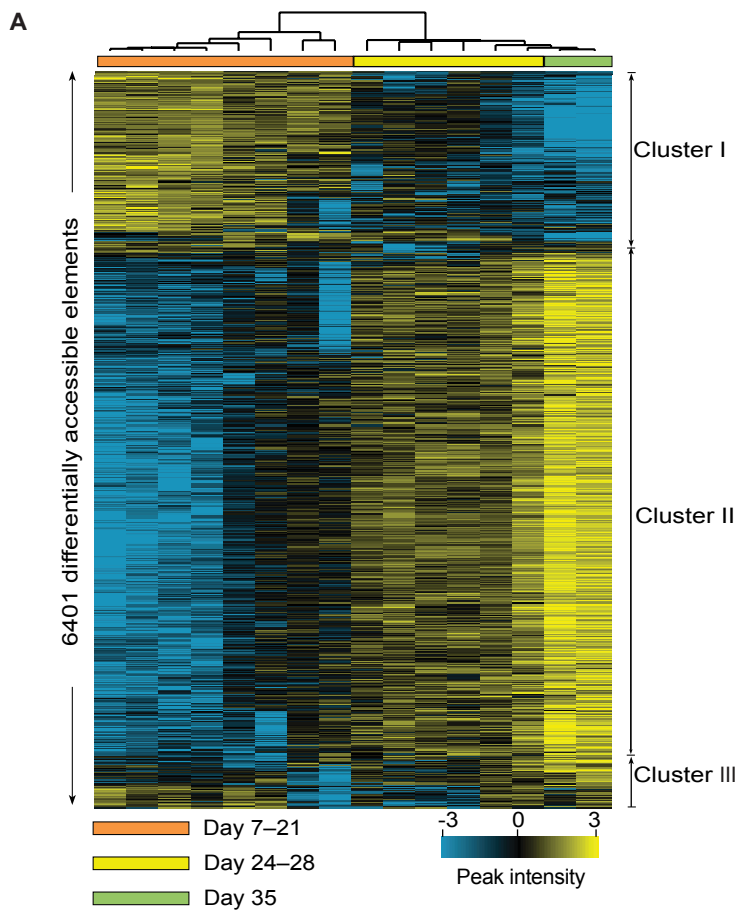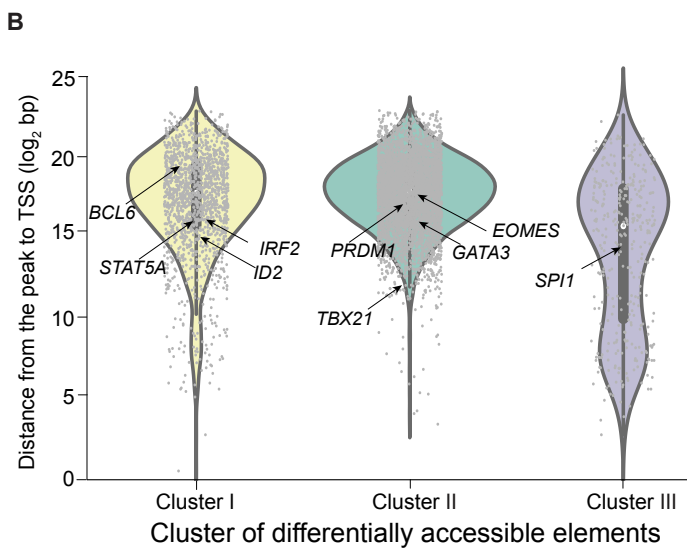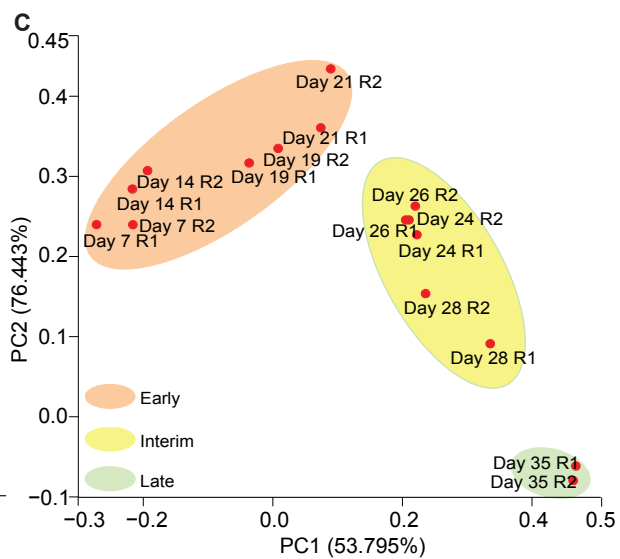

Supplement: Supplementary Figure S4 — Different stages of NK cell development have specific regulatory elements. A. Heatmap of all the 6401 differential regulatory elements in all the samples. Each column is a sample, and each row is a peak. Samples and peaks were organized by two-dimensional unsupervised hierarchical clustering. The color scale shows the relative ATAC-seq signal intensities as indicated. Top: samples at all time points were categorized into three groups, early stage: day 7–21 (orange); interim stage: day 24–28 (yellow) and late stage: day 35 (green). Samples from the same cluster are labeled with the same color. Right: differential peaks are categorized into three clusters. B. Distance from all the peaks in cluster I, II, and III to their nearest genes. Known TFs regulating NK cell differentiation are labeled. C. PCA of chromatin accessibility during NK cell differentiation. Three clusters were identified: early (day 7–21), interim (day 24–28), and late (day 35). PCA, principle component analysis. [file mmc5.pdf]

**A**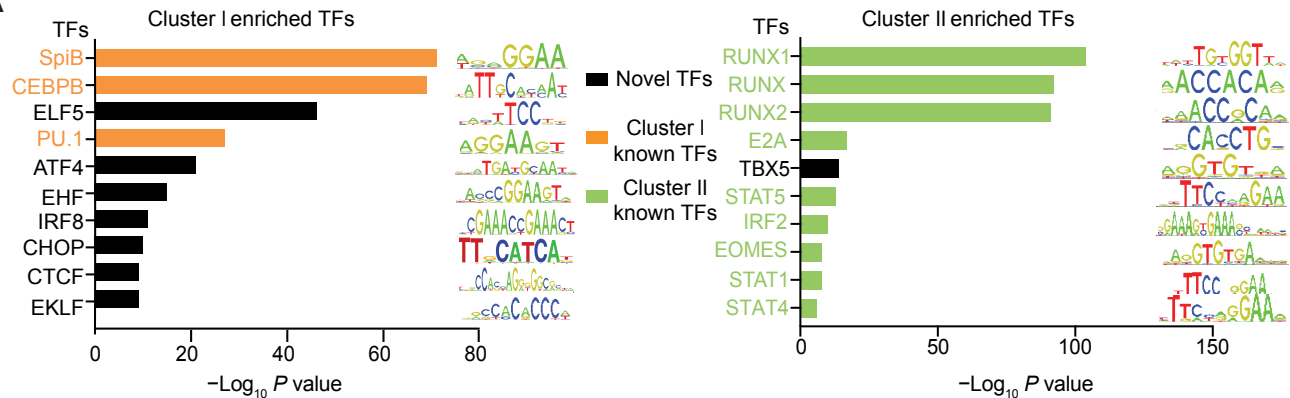**B** Expression and enriched GO terms of TF-binding genes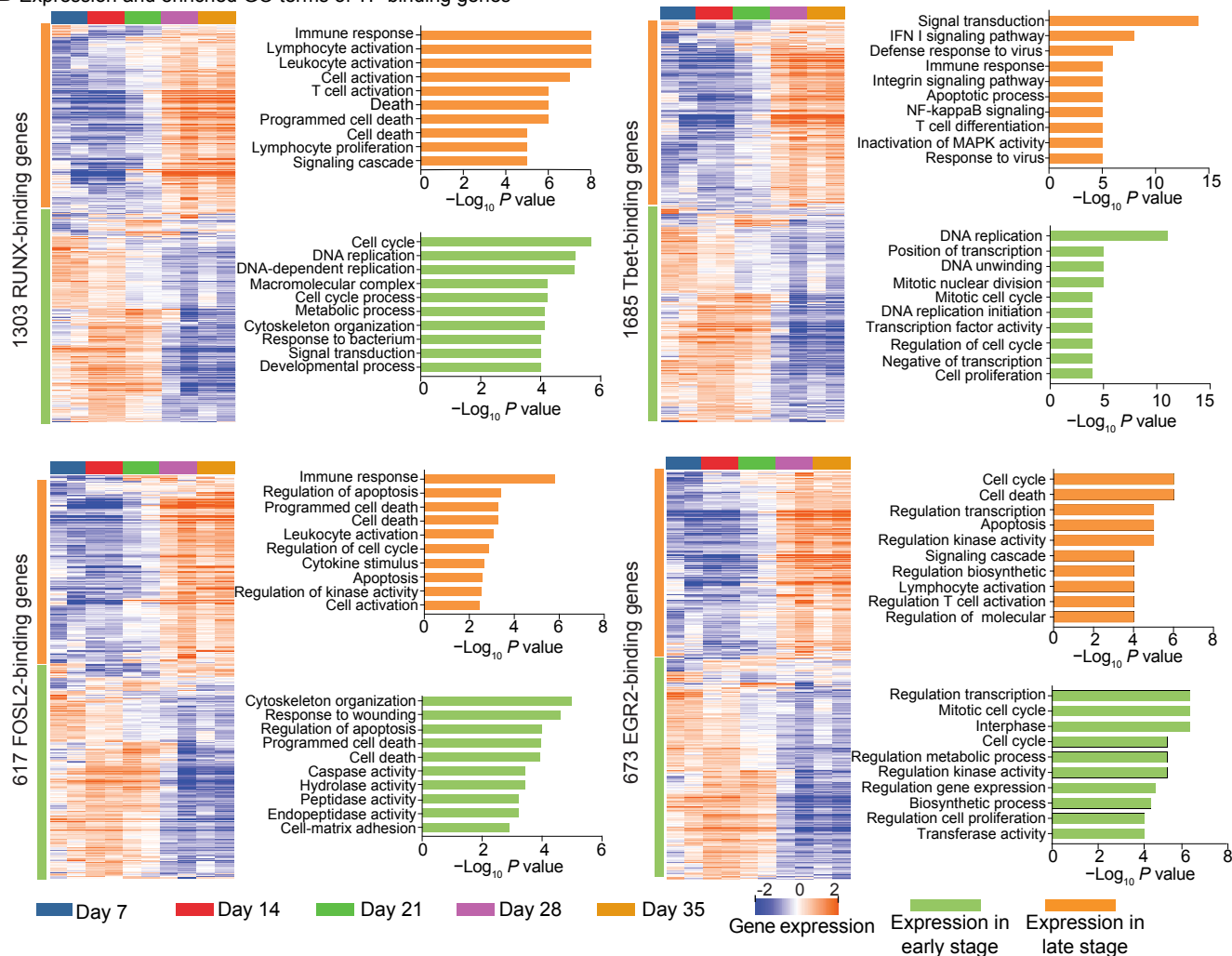

Supplement: Supplementary Figure S5 — TFs enriched at different stages of NK cell development. A. The top 10 TF motifs enriched in cluster I (left) and cluster II (right) peaks, with enrichment P values estimated from HOMER. TFs known to regulate NK cell differentiation are color-coded. B. Gene expression and enriched GO terms of TF-binding genes. Unsupervised hierarchical clustering was performed. Expression heatmap of the genes predicted to be regulated by the same TF (RUNX, T-bet, FOSL2, or EGR2) is shown on the left, whereas the top 10 most significant GO terms enriched in up-regulated (orange) and down-regulated (green) genes predicted to be regulated by each TF are shown on the left. [file mmc6.pdf]

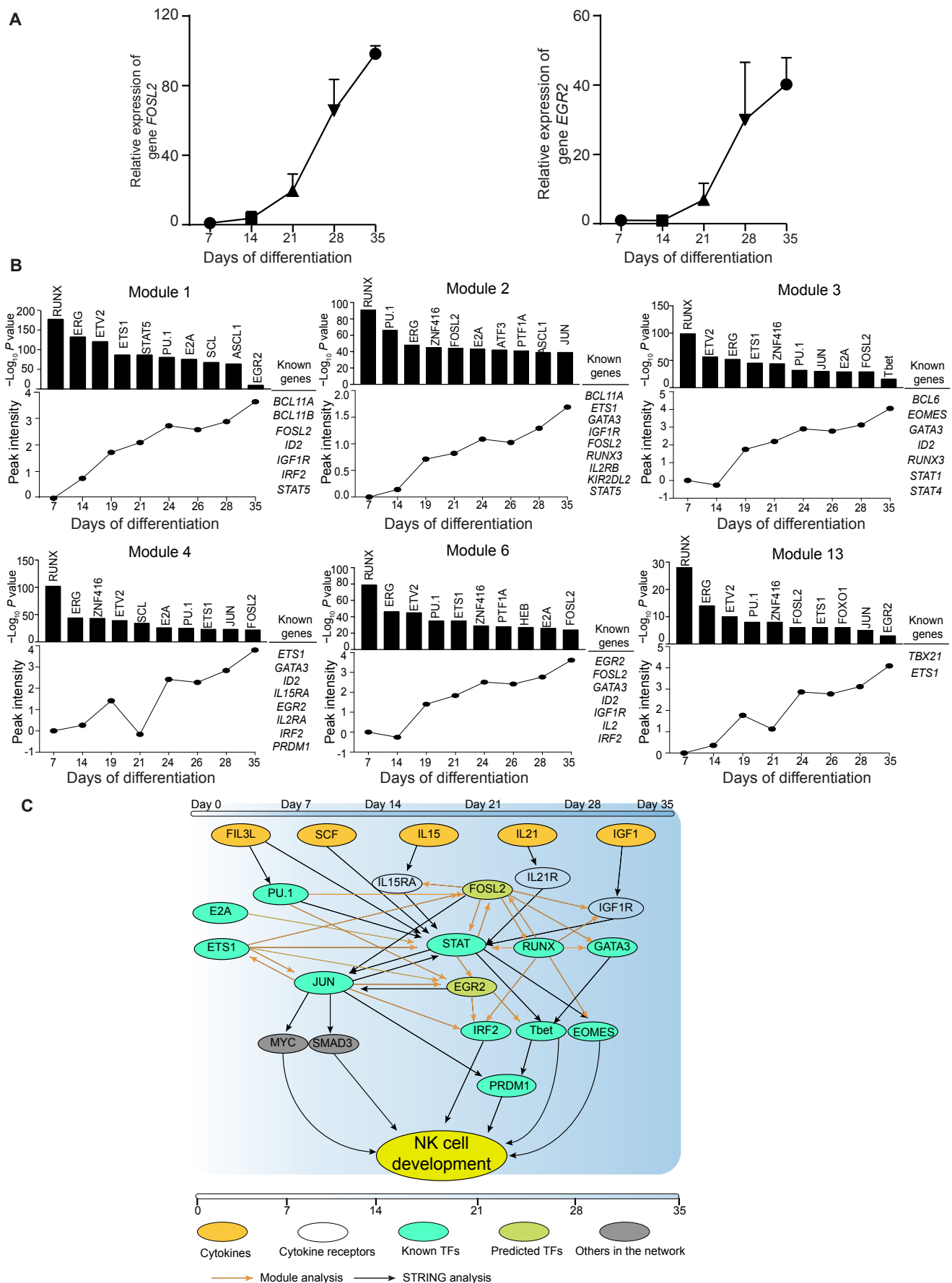

Supplement: Supplementary Figure S6 — Signaling pathways involving FOSL2 and EGR2. A. Real-time qPCR analysis (n = 3) of the genes FOSL2 (left) and EGR2 (right). Data from three replicates are presented as mean ± SEM. B. Module map analysis in Genomica: representative modules from module map analysis in Genomica. For each module, the most significantly enriched TF in the module is shown at the top, and the chromatin accessibility changes of the peaks in the corresponding module compared with day 7 are shown at the bottom. Genes associated with peaks in the respective module are listed on the right. C. Signaling pathways of the known (green circles) and predicted (olivine circles) TFs that regulate NK cell differentiation. [file mmc7.pdf]

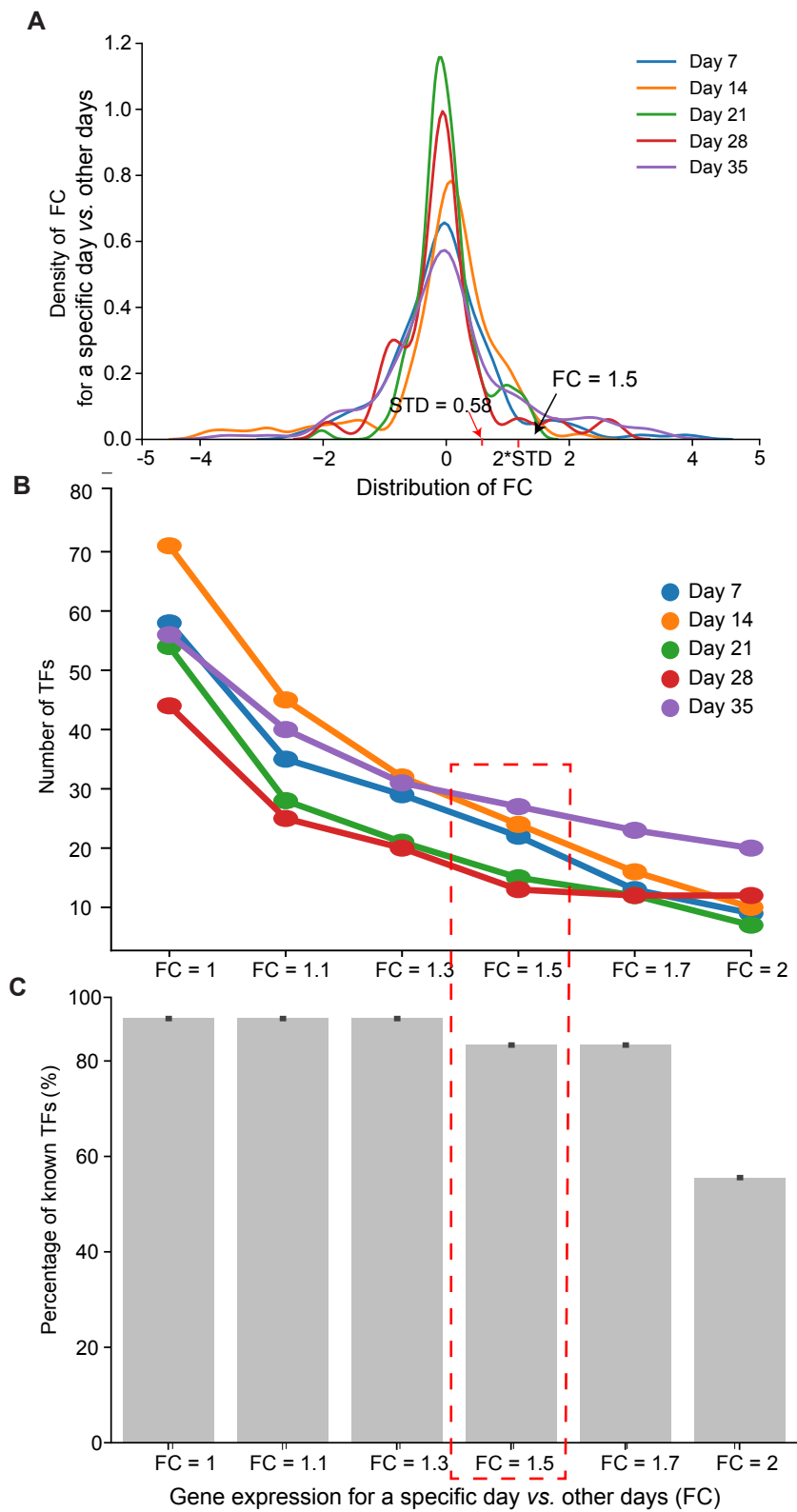

Supplement: Supplementary Figure S7 — Define time-specific TFs based on differential expression analysis. A. FC of expression distribution of all TFs at a specific day vs. other time points during NK cell differentiation. FC of 1.5 indicates 2× STD away. Time points are color coded. B. Number of TFs at different FC cutoffs. C. The ratio of known TFs at different FC cutoffs versus all known TFs. Known TFs here refer to TFs regulating NK cell development reported in the literature. FC, fold change; STD, standard deviation. [file mmc8.pdf]
